# Ivermectin-Induced Acute Psychosis in Patients Infected With COVID-19 Pneumonia

**DOI:** 10.7759/cureus.26141

**Published:** 2022-06-21

**Authors:** Lokesh Goyal, Ramesh Pandit, Trupti Pandit, Kunal Ajmera, John O Lusins, Shah Islam

**Affiliations:** 1 Hospital Medicine, CHRISTUS Spohn Hospital, Corpus Christi - Shoreline, Corpus Christi, USA; 2 Hospital Medicine, University of Pennsylvania Health Systems (Penn Medicine) / Chester County Hospital, Philadelphia, USA; 3 Pediatrics, Nemours Children's Hospital, Glen Mills, USA; 4 Epidemiology, George Washington University, Washington DC, USA; 5 Behavioral Health, Oceans Healthcare, Corpus Christi, USA; 6 Medicine, CHRISTUS Spohn Hospital, Corpus Christi - Shoreline, Corpus Christi, USA

**Keywords:** tactile hallucination, auditory hallucination, ivermectin and albendazole, covid 19, covid-19 induced psychosis

## Abstract

Coronavirus disease 2019 (COVID-19) pneumonia is an infection of the lungs that causes severe inflammation in the lungs' alveoli. It causes alveoli to fill with fluid, blood clots, and sometimes even pus. Patients who are infected with COVID-19 pneumonia experience severe cough, shortness of breath, fever, fatigue, chest pain, night sweats, chills, loss of appetite, etc. During the initial phase of the COVID-19 pneumonia pandemic, it was thought that ivermectin might be helpful in patients infected with COVID-19 pneumonia, but this was later proven to be false due to its severe risks/side effects. Infectious Disease Society of America (IDSA) suggests against the use of ivermectin for COVID-19 pneumonia. However, some providers continue to use ivermectin as one of the treatments for patients infected with COVID-19 infection. In this case report, we will discuss ivermectin causing acute psychosis in healthy 45- and 51-year-old patients with no known history of any mental health illness.

## Introduction

Ivermectin is an antiparasitic drug, approved by the FDA (Food and Drug Administration) of the United States, which is used in the treatment of diseases like ascariasis, hookworms, scabies, lice, etc. The dose of ivermectin to treat these parasitic infections are well-tolerated in human beings without much side effects. The proposed mechanism of action of ivermectin under in vitro studies shows that ivermectin inhibits the replication of SARS-CoV-2 and also competes with SARS-CoV-2 spike proteins attached to the cells of human tissue. However, the dose necessary to achieve these antiviral effects would be very high, leading to various side effects, including liver failure, altered mental status, confusion, disorientation, and even death [1,2]. We present here two case reports of a 45- and a 51-year-old patient who presented to the hospital with acute psychosis after completing high-dose of ivermectin therapy for the treatment of COVID-19 pneumonia several weeks prior.

## Case presentation

Case 1

**Figure 1:**
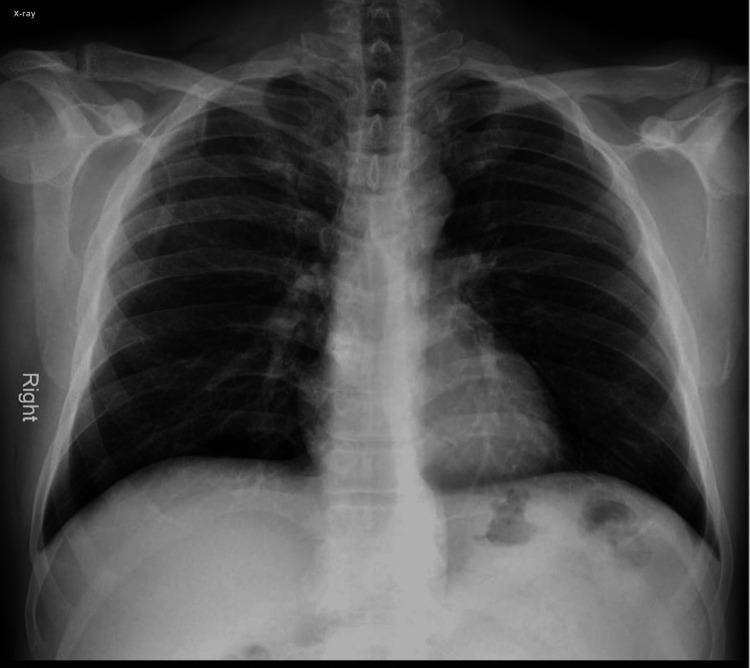
Mild COVID-19 pneumonia, but otherwise normal chest X-ray.

The patient is a 45-year-old male with a past medical history significant for only recent COVID-19 pneumonia treated outpatient at an urgent care center, who presents to the hospital with a chief complaint of auditory hallucinations and delusional behavior that has been going on for the past 24 hours. The patient states that he hears voices (without commands) and also thinks that spiders are crawling on his skin. The patient states that this has never happened before. He denies any history of psychiatric disease in him or his family. The patient's family is also in the room and agrees with the patient. The patient's family states that this is very abnormal for him. The patient works as a construction worker and reports that he recently got a COVID-19 infection and went to an urgent care center where he was prescribed ivermectin, azithromycin, and ibuprofen for treatment. The patient states that after about five days of taking the medication, he started experiencing auditory hallucinations. This change in behavior was also noticed by the family, who decided to bring the patient to the hospital. The patient denies any other past medical history. The patient smokes one pack per day, socially drinks alcohol once or twice a month, and never uses any form of recreational drugs. The patient denies any significant family history. He states that he does not take any medications at home. The patient has mild respiratory crackles in physical exam, but everything else is otherwise normal. The patient was monitored in the emergency room for 24 hours. The patient was given 1 L bolus of normal saline and an albuterol inhaler every 6 hours as needed, and the patient's outpatient medications were discontinued. The patient's symptoms started improving while in his stay in the emergency room. The patient had a chest X-ray performed in the emergency room which showed mild COVID-19 pneumonia (Figure 1).

The patient's blood test and urine drug screen were normal (Table 1). Computed tomography of the head without contrast was also negative.

**Table 1:** Blood test - Case 1.

Labs test	Results	Normal Range
Complete Blood Count (CBC)	All within normal range	Normal
Comprehensive Metabolic Panel (CMP)	All within normal range	Normal
Thyroid-Stimulating Hormone (TSH)	1 mIU/L	0.35-5.0 mIU/L
Urinary Tract infection	Negative	Negative
Urine Drug screen	Negative	Negative
Blood alcohol level	negative	negative

Inpatient psychiatry was also consulted, which recommended that the patient be transferred to an inpatient psychiatric hospital. Twenty-four hours later, the patient was transferred to the inpatient psych unit for auditory hallucinations. In the inpatient psych unit, the patient's symptoms completely resolved in about 48 hours. The patient was not prescribed any antipsychotic medication in the psych unit. The patient was then discharged from the inpatient psych unit and educated to follow up with the primary care physician (PCP)** **in the next 2 to 3 days and also a psychiatrist in two weeks. The patient was educated to discontinue taking ivermectin.

Case 2

The patient is a 51-year-old male with a past medical history of hypertension (controlled with lisinopril 20 mg PO daily) and a recent COVID-19 infection treated outpatient at an urgent care center. He presents to the hospital with the chief complaint of paranoid delusional behavior and not being himself over the last 24 hours as per his family. The patient states that he has been followed and harassed by Mexican gang members over the past one week; however, per family, this is completely untrue. The patient also made comments about being a famous movie star. The patient's family states that this is very abnormal for him, and he has never behaved like this in the past. The patient is an electrician and works full-time at his father's company. The patient's family denies any history of psychiatric disorder in him or his family. He recently had a COVID-19 infection, one week ago, with very mild symptoms of postnasal drip and fever. The patient was treated outpatient with ivermectin, azithromycin, and ibuprofen. The patient took these medications for seven days. The patient does not smoke, socially drinks alcohol once or twice a week, and never uses recreational drugs. He denies any fever, chills, night sweats, shortness of breath, chest pain, or any other issues. His physical exam is completely normal. On the evaluation of the psychiatric complaint, the patient denies any homicidal or suicidal ideation.** **The patient was monitored in the emergency room, and his blood test and urine drug screen were normal (Table 2). Computed tomography of the head without contrast was also negative. 

**Table 2:** Blood test - Case 2.

Labs test	Results	Normal Range
Complete Blood Count (CBC)	All within normal range	Normal
Comprehensive Metabolic Panel (CMP)	All within normal range	Normal
Thyroid-Stimulating Hormone (TSH)	1.8 mIU/L	0.35-5.0 mIU/L
Urinary Tract infection	Negative	Negative
Urine Drug screen	Negative	Negative
Blood alcohol level	negative	negative

Inpatient psychiatry was consulted and the patient was admitted to the inpatient psych unit. The patient's medications were discontinued except for his lisinopril. The patient's symptoms started getting better and eventually resolved 48 hours after being in the inpatient psych unit. The patient was then discharged home and educated to follow up with the primary care doctor and also psychiatrist within the next two weeks. The patient was educated to discontinue taking ivermectin.

## Discussion

Ivermectin, for over 30 years, has been used as a broad-spectrum antiparasitic medication all over the world. This drug overall has a good safety profile when used appropriately on humans and animals for its antiparasitic effects [3]. However, some studies have suggested that ivermectin has anti-inflammatory properties, and therefore this drug was extensively used during the initial stages of the COVID-19 pandemic [4,5]. Some studies have suggested that ivermectin can inhibit the alpha/beta-1 nuclear transport proteins in the host, which are responsible for intracellular transport. COVID-19 viruses hijack these proteins to enhance the infection. Ivermectin is also shown to compete with the SARS-CoV-2 spike proteins making it difficult for the virus to attach to the human cells on the tissue [6,7]. However, in vitro studies have shown that to achieve the antiviral effects of ivermectin in the human body, the dose required will be 100 times greater than that approved for human use [8,9]. 

The ivermectin mechanism of action is not very well understood, but we think that it binds to glutamate-gated chloride channels, which are mostly present in nerve and muscle cells. This increases the permeability of the nerve/muscle cells, eventually leading to the death of the parasite [10,11].

The ivermectin affects the central nervous system when it crosses the blood-brain barrier of the spinal cord and brain, therefore, blocking neuronal transmissions, which involve glutamate channels [12]. Young infants and children are at increased risk because ivermectin may easily cross their immature blood-brain barrier [13]. It also causes significant adverse reactions such as neurotoxicity (confusion, ataxia, encephalopathy, tremors, etc.) along with severe breathing issues in the treatment of onchocerciasis (dose range: 3-24 mg) [10].

Ivermectin is also associated with the posttreatment immunologic reaction, also known as the Mazzoti reaction [13], which is associated with skin rash, fever, tachycardia, hypotension, and lymphadenopathy. This reaction is usually seen in cases where ivermectin has been used for the treatment of infections like onchocerciasis, scabies, filarial parasite, etc. Symptoms of mild Mazzoti reaction typically resolve within four days; however, serious reactions can occur, especially in the treatment of filarial parasites, which can lead to coma and death. This typically happens when patients are infected with a high density of microfilariae, and when ivermectin induces microfilaricidal effects on these filariae, it causes a strong inflammatory reaction in the host tissue leading to severe local tissue damage [13].

## Conclusions

Ivermectin is an antiparasitic medication that is used worldwide and is relatively safe if used appropriately in both humans and animals. Even though ivermectin has anti-inflammatory properties, its efficacy for the treatment of COVID-19 viral pneumonia still remains a mystery. More recent clinical trials failed to show any clear evidence that ivermectin reduces on prevents COVID-19 infection. Patients who are exposed to high doses of ivermectin are at increased risk of severe side effects like psychosis, neurotoxicity, fever, tachycardia, hypotension, etc. The treatment of ivermectin-induced psychosis is to stop the offending agent and seek medical attention as soon as possible to monitor for any other adverse reactions.
